# Effect of Adjunctive Simvastatin on Depressive Symptoms Among Adults With Treatment-Resistant Depression: A Randomized Clinical Trial

**DOI:** 10.1001/jamanetworkopen.2023.0147

**Published:** 2023-02-20

**Authors:** M. Ishrat Husain, Imran B. Chaudhry, Ameer B. Khoso, Tayyeba Kiran, Nawaz Khan, Farooq Ahmad, John Hodsoll, M. Omair Husain, Haider A. Naqvi, Asad T. Nizami, Nasim Chaudhry, Hazrat A. Khan, Fareed Minhas, Jeffrey H. Meyer, Moin A. Ansari, Benoit H. Mulsant, Nusrat Husain, Allan H. Young

**Affiliations:** 1 Campbell Family Mental Health Research Institute, Centre for Addiction and Mental Health, Toronto, Ontario, Canada; 2 Department of Psychiatry, Temerty Faculty of Medicine, University of Toronto, Toronto, Ontario, Canada; 3 Department of Psychiatry, Ziauddin University, Karachi, Sindh, Pakistan; 4 Division of Psychology and Mental Health, University of Manchester, Manchester, United Kingdom; 5 Pakistan Institute of Living and Learning, Karachi, Sindh, Pakistan; 6 Department of Psychological Medicine, Institute of Psychiatry, Psychology and Neuroscience, King’s College London, London, United Kingdom; 7 Department of Psychiatry, Dow University of Health Sciences, Karachi, Pakistan; 8 Institute of Psychiatry, Rawalpindi Medical College, Rawalpindi, Pakistan; 9 Quetta Psychiatry Centre, Quetta City, Quetta, Pakistan; 10 Department of Psychiatry, Liaquat University of Medical and Health Sciences, Hyderabad, Pakistan

## Abstract

**Importance:**

Immune-metabolic disturbances have been implicated in the pathophysiology of major depressive disorder and may be more prominent in individuals with treatment-resistant depression (TRD). Preliminary trials suggest that lipid-lowering agents, including statins, may be useful adjunctive treatments for major depressive disorder. However, no adequately powered clinical trials have assessed the antidepressant efficacy of these agents in TRD.

**Objective:**

To assess the efficacy and tolerability of adjunctive simvastatin compared with placebo for reduction of depressive symptoms in TRD.

**Design, Setting, and Participants:**

This 12-week, double-blind, placebo-controlled randomized clinical trial was conducted in 5 centers in Pakistan. The study involved adults (aged 18-75 years) with a *Diagnostic and Statistical Manual of Mental Disorders* (Fifth Edition) major depressive episode that had failed to respond to at least 2 adequate trials of antidepressants. Participants were enrolled between March 1, 2019, and February 28, 2021; statistical analysis was performed from February 1 to June 15, 2022, using mixed models.

**Intervention:**

Participants were randomized to receive standard care plus 20 mg/d of simvastatin or placebo.

**Main Outcomes and Measures:**

The primary outcome was the difference between the 2 groups in change in Montgomery-Åsberg Depression Rating Scale total scores at week 12. Secondary outcomes included changes in scores on the 24-item Hamilton Rating Scale for Depression, the Clinical Global Impression scale, and the 7-item Generalized Anxiety Disorder scale and change in body mass index from baseline to week 12. C-reactive protein and plasma lipids were measured at baseline and week 12.

**Results:**

A total of 150 participants were randomized to simvastatin (n = 77; median [IQR] age, 40 [30-45] years; 43 [56%] female) or placebo (n = 73; median [IQR] age, 35 [31-41] years; 40 [55%] female). A significant baseline to end point reduction in Montgomery-Åsberg Depression Rating Scale total score was observed in both groups and did not differ significantly between groups (estimated mean difference for simvastatin vs placebo, −0.61; 95% CI, −3.69 to 2.46; *P* = .70). Similarly, there were no significant group differences in any of the secondary outcomes or evidence for differences in adverse effects between groups. A planned secondary analysis indicated that changes in plasma C-reactive protein and lipids from baseline to end point did not mediate response to simvastatin.

**Conclusions and Relevance:**

In this randomized clinical trial, simvastatin provided no additional therapeutic benefit for depressive symptoms in TRD compared with standard care.

**Trial Registration:**

ClinicalTrials.gov Identifier: NCT03435744

## Key Points

**Question:**

Does adjunctive treatment with 20 mg/d of simvastatin lead to an improvement in depressive symptoms in adults with treatment-resistant depression?

**Findings:**

In this randomized clinical trial of 150 participants with treatment-resistant unipolar depression, 12 weeks of 20 mg/d of simvastatin added to standard care did not show a statistically significant benefit compared with placebo added to standard care on the overall course of depressive symptoms.

**Meaning:**

In this study, simvastatin was not beneficial for the treatment of symptoms of treatment-resistant depression compared with standard care.

## Teaser

This randomized clinical trial of adults with treatment-resistant depression assesses whether adjunctive simvastatin therapy improves depressive symptoms compared with placebo.

## Introduction

Studies of individuals with major depressive disorder (MDD) in high-income countries and low-middle income countries (LMICs) suggest that up to one-third will not respond to first- and second-line pharmacotherapy.^1,2^ Current pharmacotherapy for treatment-resistant depression (TRD) has high rates of nonresponse, relapse, and problematic adverse effects.^3,4^ Novel, cost-effective, and efficacious treatments are needed to reduce the global burden of TRD.

Replicating evidence implicates immune-metabolic dysfunction in the pathophysiology of at least a subset of individuals with MDD.^5,6,7,8,9,10,11^ Statins or 3-hydroxy-3-methylglutaryl-coenzyme A reductase inhibitors reduce cholesterol and are used routinely to treat metabolic disorders and prevent cardiovascular or cerebrovascular disorders.^12^ In addition to their lipid-lowering properties, statins have anti-inflammatory, antioxidant, and glutamatergic effects that are neuroprotective.^13^ They also promote neuroplasticity and modulate monoamines.^13^ Given these pleiotropic effects on pathways implicated in the pathophysiology of MDD, their well-established tolerability and safety, and low costs, statins have been proposed as repurposed treatments for MDD.^13^ In a Swedish study of more than 1 million statin users, statins were associated with a reduced risk of depressive disorders even after adjustment for antidepressant use.^14^ Small pilot randomized clinical trials (RCTs)^15,16,17,18,19^ of patients with MDD suggest that statins may be safe and effective adjunctive treatments for MDD. However, to our knowledge, all the RCTs conducted to date have had small samples, preventing them from reliably assessing antidepressant efficacy, and none have focused on patients with TRD.

In this context, we conducted a multicenter, 12-week RCT to determine the efficacy and tolerability of simvastatin as an adjunctive treatment to standard care in adults with TRD. Simvastatin was selected because it is the most lipophilic statin^12^ and hence would have the greatest propensity to cross the blood-brain barrier. We hypothesized that participants randomized to adjunctive simvastatin would show more improvement in depressive symptoms than those randomized to placebo at 12 weeks.

## Methods

This study was a 2-group, placebo-controlled RCT of 20 mg/d of simvastatin plus standard care vs placebo plus standard care. The trial protocol is presented in Supplement 1. The study was conducted in outpatient psychiatric clinics in 5 urban centers in Pakistan (Hyderabad, Karachi, Lahore, Quetta, and Rawalpindi) from March 1, 2019, to February 28, 2021. In Pakistan, standard care of TRD typically consists of regular outpatient psychiatric follow-up and treatment with psychotropic medications. There is minimal access to supportive or specific psychological interventions for MDD in Pakistan.^20^ The National Bioethics Committee of the Pakistan Health Research Council approved the study and its informed consent form. All participants provided written informed consent after reading the information in English or Urdu. This study followed the Consolidated Standards of Reporting Trials (CONSORT) reporting guideline.

### Participants

Inclusion criteria were as follows: men and women aged 18 to 75 years; a diagnosis of MDD and a major depressive episode confirmed by the Structured Clinical Interview for *Diagnostic and Statistical Manual of Mental Disorders* (Fifth Edition)^21^ of which the depression module has been validated for use in the Urdu language and has been used in a previous study in Pakistan^22^; and 2 or more failed trials of antidepressant medication, at the minimum effective or higher dosage (as per the British National Formulary^23^ and Maudsley Prescribing Guidelines^24^) for at least 6 weeks. A relapse while taking an antidepressant qualified as a failed treatment trial. Participants were also required to score 14 or higher on the 24-item of the Hamilton Rating Scale for Depression (HamD-24)^25^ and to demonstrate the capacity to provide informed consent as assessed by their own clinician and the ability to complete the study assessments and take oral medication. A negative pregnancy test result and ongoing effective contraception (ie, use of a barrier method or the oral contraceptive pill) were required for women of childbearing age. All participants were of South Asian ethnicity.

Exclusion criteria were as follows: a primary psychotic disorder or bipolar disorder; history of intolerance to statins or presence of any contraindication to statins; currently taking a statin; presence of any unstable physical condition or neurological problem; presence of an autoimmune or inflammatory disorder (eg, systemic lupus erythematosus, rheumatoid arthritis, or inflammatory bowel disease); alcohol or substance use disorder within the preceding 6 months; active suicidal ideation; pregnant or breastfeeding; serum low-density lipoprotein cholesterol (LDL-C) level of less than 80 mg/dL (to convert to milligrams per deciliter, multiply by 0.0259) at baseline; and elevated aspartate transaminase, alanine transaminase, lactate dehydrogenase, or creatine phosphokinase level at baseline.

During the 12-week trial, participants were required to continue to take a stable dose of the antidepressant they were currently taking and not to start taking a new antidepressant or engaging in a new psychosocial intervention. However, those who were already engaged in a psychosocial intervention at the screening stage were permitted to continue it.

### Randomization and Masking

Randomization was conducted by an independent statistical support service based in the United Kingdom. Randomization was stratified by severity of depressive symptoms at baseline and study center. Participants were randomized using a computer-generated, random permuted block method with variable block sizes. Allocation was masked from study investigators, assessors, participants, and their families or caregivers. All participants were assigned a unique study identification number once they provided informed consent and eligibility was confirmed. A central trial pharmacy prepared a 12-week package of 20 mg/d of simvastatin or placebo, using identical tablets. The packages bearing the participant’s identification number were sent to the site pharmacy, which was also blinded to treatment allocation.

### Clinical and Research Procedures

Simvastatin was started at a dosage of 20 mg/d. Assessments were conducted at baseline and weeks 2, 4, 8, and 12. Study medication was dispensed at each study visit, at which point research assistants conducted pill counts to assess adherence. We also used the 4-item Morisky Medication Adherence Scale.^26^

### Assessments

The primary outcome was differences in Montgomery-Åsberg Depression Rating Scale (MADRS)^27^ scores at week 12 between the 2 treatment groups. Secondary outcomes included rates of response and remission, with response defined as 50% or greater reduction in MADRS scores and remission defined as a MADRS score of 10 or less at week 12. Other outcomes were scores on the HamD-24,^25^ Clinical Global Impression scale,^28^ and the 7-item Generalized Anxiety Disorder scale^29^; all these instruments have been validated for use in the Urdu language and have been used in a previous study in Pakistan.^30^ Although our published study protocol^31^ proposed to use the UKU Side Effect Rating scale^32^ to assess adverse events (AEs), the trial steering committee deemed this approach too burdensome for participants and recommended changing to a more specific AE scale. Thus, AEs were assessed using a 30-item checklist based on the product characteristics of simvastatin.

#### Immune Metabolic Parameters

Body mass index (BMI; calculated as weight in kilograms divided by height in meters squared) was assessed at baseline and week 12. Participants were also requested to provide 2 blood samples (at baseline and at week 12). This was optional and did not influence recruitment to the study. Biomarkers analyzed included C-reactive protein (CRP), quantified using Spinreact CRP Latex Agglutination Test, and plasma lipids (high-density lipoprotein cholesterol [HDL-C] and LDL-C).

### Interrater Reliability

To assess interrater reliability for the primary outcome measure, video recordings of MADRS interviews were coded independently by site raters. The intraclass correlation coefficient among the 5 main research assistants was 0.753, reflecting good interrater reliability.

### Sample Size Determination

The study was powered assuming a priori that an effect size (ES) of 0.60 or larger would be clinically meaningful, congruent with evidence from a study^33^ suggesting that an ES of 0.40 or larger provides clinical utility. Thus, the sample size was calculated based on a group standardized mean difference of 0.60 on the MADRS for simvastatin, adjusted for a 20% attrition rate. With these assumptions, a sample size of 150 would give 90% power to detect a standardized mean difference of 0.60 or larger with a level of significance set at α = .05. This first power calculation did not include the gain in statistical efficiency from adjusting for baseline variables.^34^ For a given sample size, the gain (or design effect) is approximately equal to 1 – ρ^2^, where ρ is the correlation between baseline and outcome. In addition, a correction factor of 1 per group is added to the initial sample size before the design effect.^35^ Thus, a ρ of 0.50 (as shown for patient-reported outcomes^34^) and a sample size of 150 would yield an effective sample size of 202 (n = 162 with 20% attrition), allowing us to detect an ES of 0.51.

### Statistical Analysis

Statistical analysis was performed from February 1 to June 15, 2022. Initial descriptive analysis was conducted to characterize the randomized groups at baseline on main demographic characteristics and clinical measures. No statistical tests were used to compare groups. The main analysis followed the intention-to-treat principle. For the primary hypothesis, the MADRS scores at week 12 were compared between groups using a mixed-effect model. The outcome for the model was postbaseline MADRS scores, and the fixed effects included baseline MADRS scores, treatment group, randomization site, time (postbaseline time points), and a treatment group × time point interaction. To account for the dependencies between repeated measures on the same participants, an intercept for each participant was included as a random effect.

A linear contrast was used to test the primary hypothesis: the baseline adjusted difference in MADRS scores between groups at week 12. Two-tailed tests and an α = .05 were used for significance. All estimates are presented with 95% CIs. To estimate ES, standardized regression coefficients were calculated using the overall SD of the baseline outcome scores as the metric.

Secondary analyses used similar mixed models, with baseline adjusted with time, treatment group, and the treatment group × time interaction as independent variables. As for the primary outcome, the main time point of interest was 12-week follow-up, and treatment effects were determined by contrasts at the measured time point. To compare remission rates between groups, logistic regression was used in which participants were classified as being in remission or not. Outcomes at time points other than 12-week follow-up were considered exploratory, and the focus was ES (ie, standardized regression coefficients).

An exploratory analysis was conducted to assess whether baseline BMI, CRP, or lipids moderated response to simvastatin by adding each baseline measure to a model for the primary outcome at 12 weeks, adjusted for baseline MADRS scores and including a treatment group × baseline BMI, CRP, and lipids interaction. Lipids, BMI, and CRP were considered moderators if the interaction with treatment group was significant (at a significance level of α = .05, using bootstrapping for inference and construction of CIs), in which case exploratory plots that assess the group effect at different levels of the moderators were used to study the nature of the moderation.

To assess whether changes in peripheral levels of CRP and lipids mediate the effect of the intervention on the change in MADRS scores, an initial descriptive analysis was conducted to assess bivariate association between treatment group and change in CRP and lipids and between change in CRP and lipids (at 12 weeks) and change in MADRS scores. A mediation model was then fitted to the data that jointly modeled the effects of treatment on the change in CRP and lipids at 12 weeks and on MADRS scores at 12 weeks. The mediation effect was tested by the proportion of the total effect from treatment group to change in MADRS attributable to changes in CRP and lipids. This model was fitted in Lavaan in R software, version 4.1.2 (R Foundation for Statistical Computing) and the indirect effect estimated through bootstrap resampling.

To compare the frequency of AEs, we visualized percentage risk and relative risk ratios for each AE and present them by treatment group. To handle missing data, we identified baseline factors associated with missingness using logistic regression with the outcome (missing or not). Two variables were associated with missingness and were thus included in the analysis models, namely years of education and time since first diagnosis in months. Mixed-effect models fitted through maximum likelihood were conducted, assuming the data to be missing at random.

## Results

**Figure 1.**
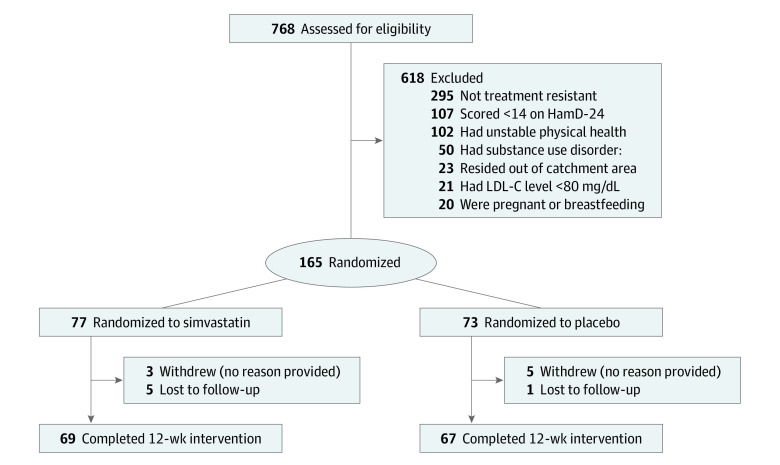
Flow Diagram HamD-24 indicates 24-item of the Hamilton Rating Scale for Depression; LDL-C, low-density lipoprotein cholesterol (to convert to mmol/L, multiply by 0.0259).

A total of 150 participants were randomized to simvastatin (n = 77; median [IQR] age, 40 [30-45] years; 43 [56%] female and 34 [44%] male) or placebo (n = 73; median [IQR] age, 35 [31-41] years; 40 [55%] female and 33 [45%] male). Of the 150 participants included in the intention-to-treat analysis, at the end of the study, 8 (5%) withdrew (3 randomized to simvastatin and 5 to placebo) and 6 (4%) were unavailable for follow-up. Figure 1 shows the flow of participants up to and including the 12-week visit. Baseline data for participants in each group are presented in Table 1. The median duration of current major depressive episode was 5 months (IQR, 3-10 months) in the placebo group and 5 months (IQR, 3-7 months) in the simvastatin group. All participants were prescribed an antidepressant; most were also prescribed adjunctive antipsychotic, mood-stabilizing, or anxiolytic agents (Table 1); and none were receiving any psychotherapy during the trial. Baseline MADRS scores were similar in both groups and were in the moderate to severe range. Other outcome measures were also similar between groups at baseline. Baseline CRP and lipid levels were unremarkable (Table 1).

**Table 1.** Baseline Demographic and Clinical Characteristics of 150 Randomized Participants^a^

Characteristic	Placebo group (n = 73)	Simvastatin group (n = 77)
Age, median (IQR), y	35 (31-41)	40 (30-45)
Sex at birth		
Male	33 (45)	34 (44)
Female	40 (55)	43 (56)
Educational level, median (IQR), y	8.0 (3.0-10.0)	8.0 (0.0-10.0)
Marital status		
Single	14 (19)	9 (12)
Married	56 (77)	62 (81)
Divorced or widowed	3 (4)	6 (8)
Household status		
Lives alone	33 (45)	43 (56)
Lives with family members	40 (55)	34 (44)
No. in family, median (IQR)	7 (5-10)	7 (5-9)
Household income, median (IQR), Rs	25 000 (16 000-40 000)	25 000 (16 000-40 000)
Socioeconomic status		
Lower	40 (55)	42 (55)
Middle or upper	33 (45)	35 (45)
BMI, median (IQR)	27.4 (24.9-30.6)	26.7 (22.7-30.1)
Height, median (IQR), m	1.57 (1.52-1.65)	1.63 (1.52-1.65)
Smoker		
No	62 (85)	67 (87)
Yes	11 (16)	10 (13)
Blood pressure, median (IQR), mm Hg		
Systolic	120 (120-130)	120 (118-130)
Diastolic	80 (80-90)	80 (70-80)
No. of psychiatric hospitalizations		
0	52 (71)	62 (81)
1	13 (18)	9 (12)
≥2	8 (11)	6 (7.8)
Duration of current depressive episode, median (IQR), mo	5 (3-10)	5 (3-7)
No. of psychotropic medications		
1	25 (34)	30 (39)
2	30 (41)	33 (43)
3	16 (22)	13 (17)
4	2 (3)	1 (1)
MADRS score, median (IQR)	29 (24-34)	27 (23-33)
HamD-24 score, median (IQR)	31 (26-34)	30 (27-34)
GAD-7 score, median (IQR)	14 (12-16)	13 (11-15)
MMAS-4 score		
0	46 (63)	42 (55)
1	11 (15)	14 (18)
2	8 (11)	10 (13)
3	3 (4)	4 (5)
4	5 (7)	7 (9)
CRP, median (IQR), mg/L	1.39 (1.39-1.87)	1.39 (1.39-1.76)
Cholesterol, median (IQR), log mg/dL		
HDL-C	3.78 (3.69-3.85)	3.76 (3.69-3.83)
LDL-C	4.91 (4.66-5.04)	4.84 (4.68-4.99)

Abbreviations: BMI, body mass index (calculated as weight in kilograms divided by height in meters squared); CRP, C-reactive protein; GAD-7, 7-item Generalized Anxiety Disorder Scale; HamD-24, 24-item Hamilton Rating Scale for Depression; HDL, high-density lipoprotein cholesterol; LDL, low-density lipoprotein cholesterol; MADRS, Montgomery-Åsberg Depression Rating Scale; MMAS-4, Morisky Medication Adherence Scale (4-item); R, rupees.

SI conversion factors: To convert cholesterol to milligrams per deciliter, multiply by 0.0259; and CRP to milligrams per liter, multiply by 10.

^a^ Data are presented as number (percentage) unless otherwise indicated.

**Figure 2.**
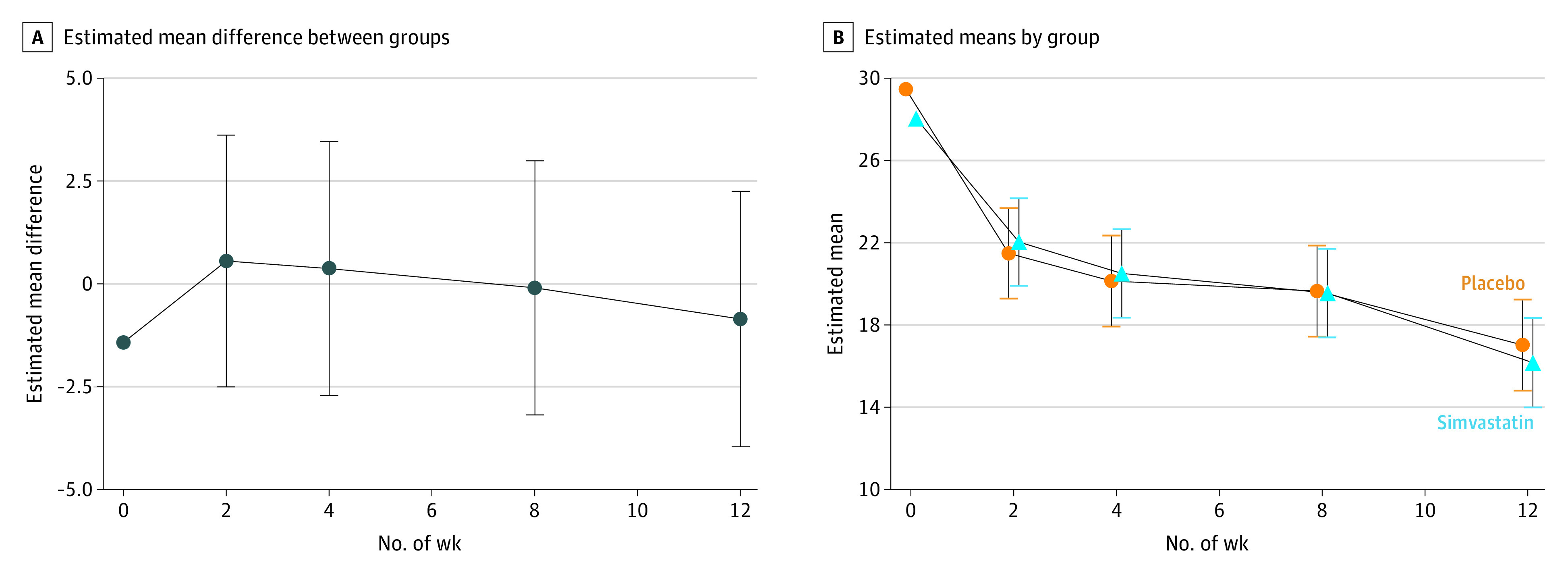
Trajectory of Montgomery-Åsberg Depression Rating Scale Scores Whiskers indicate 95% CIs.

There was no evidence of treatment effects of simvastatin at 12 weeks because MADRS scores did not differ significantly between groups (estimated mean difference, −0.61; 95% CI, −3.69to 2.46; standardized ES, −0.08; 95% CI, −0.51 to 0.34; *P* = .70) (Table 2 and Figure 2). In the exploratory moderator analysis, treatment effects at 12 weeks did not differ according to baseline BMI, MADRS score, CRP level, or lipid levels (except for LDL-C) (eTables 1-5 in Supplement 2): effects of simvastatin on MADRS scores at 12 weeks were not moderated by baseline BMI (difference in slopes [b] = −0.09; 95% CI, −0.70 to 0.51; *P* = .77), MADRS (b = −0.31; 95% CI, −0.74 to 0.11; *P* = .15), CRP (b = −2.92; 95% CI, −5.91 to 0.08; *P* = .06), or HDL-C (b = 1.54; 95% CI, −1.72 to 1.48; *P* = .35). Higher baseline LDL-C level was significantly associated with lower 12-week MADRS scores in the placebo group but not the simvastatin group (b = −4.82; 95% CI, −7.77 to −1.87; *P* = .001) (eFigure 1 in Supplement 2). Analysis of secondary outcomes revealed similar findings to those of MADRS scores, with scores on the other outcome measures (HamD-24, 7-item Generalized Anxiety Disorder scale, and Clinical Global Impression) improving similarly in both treatment groups (Table 3 and eFigure 3 in Supplement 2). Rates of response were equivalent in both groups at 38%, and rates of remission were similar in the simvastatin (23%) and placebo group (22%). There were no significant differences in odds of remission between groups (Table 3).

**Table 2.** Montgomery-Åsberg Depression Rating Scale Scores by Treatment Condition and Time Point

Variable	Baseline	Week 2	Week 4	Week 8	Week 12
Descriptive statistics					
Simvastatin					
No. of patients	77	73	71	71	69
Mean (SD) HamD-24 score	28.0 (7.2)	21.9 (9.2)	20.3 (8.5)	19.3 (10.2)	15.5 (7.4)
Placebo					
No. of patients	73	68	67	67	67
Mean (SD) HamD-24 score	29.5 (7.2)	21.8 (10.2)	20.4 (9.7)	19.8 (9.4)	17.2 (11.1)
Adjusted group difference					
Mean (95% CI)	NA	0.71 (−2.32 to 3.74)	0.57 (−2.49 to 3.62)	0.13 (−2.92 to 3.19)	−0.61 (−3.69 to 2.46)
SES (95% CI)	NA	0.1 (−0.32 to 0.52)	0.08 (−0.34 to 0.5)	0.02 (−0.4 to 0.44)	−0.08 (−0.51 to 0.34)
*t* (*df*)	NA	0.5 (339.3)	0.4 (343.8)	0.1 (344)	−0.4 (348.7)
*P* value	NA	.65	.72	.93	.70

Abbreviations: HamD-24, Hamilton Rating Scale for Depression; NA, not applicable; SES, standardized effect size.

**Table 3.** Estimated Mean Group Differences Between Simvastatin and Placebo and Odds Ratios for the Secondary Outcomes

Measure	Estimate (95% CI)^a^	SMD (95% CI)	*P* value
MADRS remission	0.89 (0.28 to 2.8)	NA	.84
HamD-24	−0.39 (−3.29 to 2.5)	−0.06 (−0.51 to 0.39)	.79
GAD-7	−0.57 (−1.98 to 0.85)	−0.09 (−0.31 to 0.13)	.44
CGI	0.66 (0.31 to 1.43)	NA	.29
MMAS-4	0.10 (−0.18 to 0.38)	0.08 (−0.14 to 0.32)	.48
BMI	0.12 (−0.06 to 0.3)	0.02 (−0.01 to 0.06)	.18

Abbreviations: BMI, body mass index; CGI, Clinical Global Impression Scale; GAD-7, 7-item Generalized Anxiety Disorder Scale; HamD-24, 24-item Hamilton Rating Scale for Depression; MADRS, Montgomery-Åsberg Depression Rating Scale; MMAS-4, 4-item Morisky Medication Adherence Scale; NA, not applicable; SMD, standard mean difference.

^a^ Estimates are odds ratios for MADRS remission and CGI and mean differences for all other measures.

In the mediation analysis, plasma CRP, HDL-C, and LDL-C levels were unaffected by treatment and thus showed no evidence of effects of changes in these measures on MADRS scores (eTables 6-8 in Supplement 2). There were no serious adverse events, and the frequency of AEs did not differ significantly between treatment groups (eFigure 2 in Supplement 2). In a post hoc secondary analysis, we found that the number of baseline psychotropic medications did not impact the estimates of the treatment effect. Finally, pill counts conducted at each study visit and scores on the Morisky Medication Adherence Scale indicated good adherence to both study drugs (Table 2).

## Discussion

This RCT found that the antidepressant effect of 20 mg/d of adjunctive simvastatin for 12 weeks did not differ from the effect of placebo in Pakistani adults with TRD. To our knowledge, this is the largest RCT of adjunctive statins in MDD to date and the first in TRD. In a meta-analysis^19^ of 4 small RCTs^15,16,17,36^ of adjunctive statins in MDD, statins were associated with a medium antidepressant ES compared with placebo, with no differences in acceptability, tolerability, and safety. Sample sizes in previous trials were smaller than our trial, ranging from 48 to 90 participants. More importantly, none of these prior trials^15,16,17,36^ focused on TRD. The smaller samples and differences in participants recruited in these prior RCTs and ours may in part account for their conflicting results.

However, our negative results are congruent with an RCT^36^ of 90 participants with MDD aged 15 to 25 years, who were randomized to standard care plus 10 mg/d of rosuvastatin or placebo. That trial reported no significant antidepressant effect of adjunctive rosuvastatin on the MADRS after 12 weeks. In an exploratory analysis, the response to rosuvastatin was significantly higher in participants 18 years and younger and in those with higher depressive symptom severity at baseline.^36^ Because our sample was restricted to participants older than 18 years, we cannot assess the effect of adjunctive simvastatin in those 18 years or younger, but we found no moderating effect of depression severity at baseline. Future trials of statins in TRD may consider enriching their samples with younger participants.

### Strengths and Limitations

Our study has both strengths and limitations. The relatively large sample size and high retention rates (93% overall) are strengths of this study; we had the power to detect a medium antidepressant ES for simvastatin. The pragmatic design and low-resource, community setting are additional strengths of our trial. Most RCTs of pharmacotherapies, including antidepressants and statins, are conducted in high-income countries, limiting their international representativeness.^37,38^ Randomized clinical trials of psychopharmacotherapy conducted in LMICs, such as Pakistan, are needed to identify unexpected harms that can be caused by extrapolating the results of RCTs from populations of high-income countries to those in LMICs.^39^ Conversely, Pakistan is an LMIC where diet, nutritional status, and other lifestyle factors, such as exercise and potential exposure to immune-related illness, differ from those in high-income countries. Given this study’s sample size, it is expected that these factors were balanced across both groups.

This study also has some limitations. The use of standard care is a potential limitation of our study. However, given the severity of MDD in the TRD sample recruited, it would have been challenging to standardize treatment in our community outpatient participants. Although most participants were receiving combination treatments involving antidepressants, mood stabilizers, atypical antipsychotics, and anxiolytic medications, these pharmacotherapies were similar across groups throughout the trial. Furthermore, we conducted a post hoc secondary analysis and found that the number of baseline psychotropic medications did not impact the estimates of the treatment effect.

As in most previously published RCTs of mood disorders, our placebo group experienced a large antidepressant response. We have previously suggested that the nonspecific therapeutic effects of attentive measurement-based care through repeated study visits in a clinical trial contribute to the placebo response,^40^ particularly in low-resource settings such as Pakistan.^41^ However, the placebo effect in the current trial was similar to the pooled placebo effect in clinical trials for TRD conducted across high- and low-income countries.^42^ Other factors contributing to the placebo response specific to our study include the relatively low minimal symptom severity required for inclusion into the trial (ie, an HamD-24 score of 14), which may have led to regression to the mean in some participants.^43^

## Conclusions

In this RCT of simvastatin added to standard care in the short-term treatment of TRD, adjunctive simvastatin provided no therapeutic benefit compared with placebo added to standard care. Further research is needed to identify immune-metabolic phenotypes of depression that may be more responsive to, or preventable with, targeted statins or other immunomodulatory treatments.

## Notes

Supplement 1.
Trial Protocol

Supplement 2. **eTable 1.** Mean Group Difference Between Slopes for Baseline MADRS Scores per Visit **eTable 2.** Mean Group Difference Between Slopes for Baseline BMI Scores per Visit **eTable 3.** Mean Group Difference Between Slopes for Baseline CRP Scores per Visit **eTable 4.** Mean Group Difference Between Slopes for Baseline HDL Scores per Visit **eTable 5.** Mean Group Difference Between Slopes for Baseline LDL Scores per Visit **eTable 6.** Estimated Means and 95% CI for Mediation Model With CRP (at 12 Weeks) as Mediator **eTable 7.** Estimated Means and 95% CI for Mediation Model With log(HDL) (at 12 Weeks) as Mediator **eTable 8.** Estimated Means and 95% CI for Mediation Model With log(LDL) (at 12 Weeks) as Mediator **eFigure 1.** Estimated Mean Slopes and 95% CI for Baseline (Log) LDL vs Outcome by Group and Study Visit **eFigure 2.** Frequency of Adverse Effects Across Treatment Groups **eFigure 3.** Clinical Global Impression Outcomes Across Treatment Groups

Supplement 3.
Data Sharing Statement
