# Supplementary material for: Effect of Adjunctive Simvastatin on Depressive Symptoms Among Adults With Treatment-Resistant Depression: A Randomized Clinical Trial
Source: JAMA Netw Open. 2023 Feb 20;6(2):e230147. doi: 10.1001/jamanetworkopen.2023.0147 (PMC9941891; doi:10.1001/jamanetworkopen.2023.0147)
Supplement: Supplement 1. — Trial Protocol [file jamanetwopen-e230147-s001.pdf]

Adjunctive Simvastatin for Treatment-Resistant Depression: Study Protocol of a 12-Week Randomised Controlled Trial

Version 1.1

Dr. Muhammad Ishrat Husain

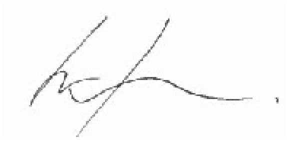

Principal Investigator

Background

Major depressive disorder (MDD) is a leading source of disability worldwide [1]. At least a third of people with MDD fail to respond to an adequate trial with first-line antidepressants and hence have varying degrees of treatment-resistant depression (TRD) [2, 3]. Treatment resistance significantly contributes to the global burden of depression [4]. Relatively few pharmacological treatments are currently available for TRD [4]. Therefore, the investigation of mechanistically novel neurotherapeutic targets (i.e. beyond standard monoamine-based approaches) for TRD is a clear unmet need in the field [4]

Statins are pleiotropic agents and accumulating evidence from preclinical studies has suggested that these agents may have antidepressant properties [4, 5]. A recent population-based study indicated that concomitant use of statins and selective serotonin reuptake inhibitors (SSRIs) resulted in significantly less psychiatric hospital contacts due to depression compared to people who used SSRIs alone [6]. Moreover, a meta-analysis of population-based studies has suggested that statin use may decrease the risk of incident depression [7]. In addition, a recent meta-analysis of three small randomised controlled studies (RCTs) have provided evidence that adjunctive treatments with statins could be efficacious for treatment of depressive symptoms in patients with moderate-to-severe depression [8].

Several mechanisms may contribute to the putative antidepressant mechanisms of statins including anti-inflammatory, anti-oxidant, neurotrophic and even monoamine-based effects. [5, 9-11] Aberrations in these pathways have been increasingly implicated in the neurobiology of depression [12-14]. Moreover, abnormalities in lipid metabolism have also

been implicated in the pathophysiology of depression, specifically for the development of atypical depressive symptoms i.e. increased appetite and/or weight gain, hypersomnia, leaden paralysis, fatigue and interpersonal sensitivity [15, 16]. Hence lipid-lowering effects of statins may also play a role in putative antidepressant effects of this class of drugs. Data from the STAR*D trial indicate poorer treatment response to SSRIs in those with atypical depression and therefore, statins could provide genuinely novel therapeutic targets for this group of people with TRD [17].

We plan to carry out an RCT involving 150 participants with treatment-resistant depression, with the aim of determining whether the addition of simvastatin (20mg daily), a lipophilic statin that readily crosses the blood-brain barrier [18, 19], to treatment as usual (TAU) for 12 weeks leads to a reduction in depressive symptoms compared with placebo added to TAU. We predict that treatment-resistant depression patients will show improvement in mood with simvastatin treatment and that the response will be associated with reduction in lipid biomarkers (i.e. plasma LDL and LDL/HDL ratio). A secondary hypothesis relates to simvastatin's effects on putative inflammatory biomarkers. High LDL and low HDL contribute substantially to immunological up-regulation in the development of atherosclerosis. Pro-inflammatory cytokine (Interleukin-6 and Tumour Necrosis Factor) levels are often altered in depressed patients and decreased by successful antidepressant therapy [20, 21]. We therefore predict that in those treatment-resistant depression patients who show improvement in mood with simvastatin treatment, that response will be associated with reduction in inflammatory biomarkers. Our mechanistic hypothesis is that the anti-inflammatory and lipid-reducing effects of simvastatin will improve neuroinflammation and thus mediate a response to simvastatin.

Methods

A pragmatic multi-centre, 12-week, double blind, placebo controlled randomised trial of simvastatin added to treatment as usual (TAU) for patients suffering from a DSM-5 major depressive episode that has failed to respond to at least two trials of antidepressants. This will be a parallel group study with 75 participants in each arm. Recruitment will occur at outpatient psychiatric clinics in Karachi, Lahore, Hyderabad, Rawalpindi and Quetta Pakistan. Consistency of procedures across sites will be ensured through vigorous training and supervision of researcher staff. This will include; ensuring research team members involved in the data collection and recording are centrally and consistently trained in the correct processes, having checklists for tasks involved in data collection, providing all researchers involved in assessments clear, written instructions through a Manual of Operations and Procedures (MOP) / Standard Operating Procedures (SOPs), which will detail operational data definitions, recruitment, screening, enrolment, randomization, follow-up procedures, data collection methods, data flow, case report forms (CRFs), and quality control procedures.

Participants will be randomised to receive either simvastatin and treatment as usual (TAU), or placebo and TAU for 12 weeks. All participants will provide written informed consent after reading the information in English and/or Urdu. Treatment as usual (TAU) will comprise of medication including antidepressants, mood stabilisers and antipsychotics and routine input from mental health services. There are little, if any provisions for psychosocial interventions to patients with depressive disorders in Pakistan. Simvastatin added to TAU will be 20mg taken once daily. This dose has been found to be safe and well tolerated as well as effective in reducing depressive symptoms in a population with moderate to severe depression [22].

The trial has been registered with Clinicaltrials.gov in (ClinicalTrials.gov identifier: NCT03435744).

Sample Size

We estimate a moderate effect size of 0.60 will be reported at week 12. Assuming a type-1 error of 5%, we will need to follow up 120 patients across the two groups to achieve 90% power at Week 12. With an anticipated dropout rate of 20% by Week 12, we will randomise 150 participants. An effect size of 0.4 or greater for difference in MADRS scores in randomised controlled trials of antidepressants versus placebo has been suggested as necessary to suggest clinical utility for an antidepressant used as monotherapy. A sample of 120 participants is therefore powered to detect moderate to large effect sizes and trends towards significance in areas of smaller effect.

Study Procedure

Recruitment

Initially, the research team will approach local clinical teams to inform them about the trial and provide them with the inclusion and exclusion criteria. They will then ask psychiatrists in each outpatient department if they are able to identify any patients who may be potentially eligible to take part in the study. The consultant psychiatrist will briefly explain the study to the patient and if the patient agrees, consultants will refer patients to the research team.

Researcher staff will work closely with the clinical team to determine if patients are suitable to participate in the trial. If patients meet the inclusion criteria, and the consultant psychiatrist and clinical team agree that they could be potential participants, the research assistant (RA) will arrange an appointment to explain the study verbally and provide them with the participant information sheet. The patient will be given at least 24 hours to read and understand this leaflet. If after this they decide that they are willing to take part, an appointment will be arranged to obtain signed informed consent for trial participation and also signed consent for access to their medical notes. Literate participants will sign the consent forms but, if the participant cannot read and sign the consent form, his/her caregiver and/or an independent person who the participant agrees to be a witness, will be requested to read the participant information leaflet and consent form to patient. Participants will then be asked to place a thumbprint on the consent form if they agree to participate, which will be countersigned by a witness/caregiver.

Screening

At the first visit, participants will undergo structured diagnostic interviews using the Structured Clinical Interview for DSM-5 (SCID-5) to confirm a diagnosis of DSM-5 current major depressive episode [23]. This tool has been validated for use in the Urdu language and has been used in previous studies in Pakistan [24]. The 24-item Hamilton Rating Scale for Depression (HRSD-24) will be used to assess severity and a score $\geq$14 will be used as the minimum threshold for study entry [25].

All patients must currently be on an antidepressant and must have had a non-response to $\geq$2 oral antidepressant treatments in the current episode (including the one they are currently

taking). The ≥2 antidepressants must have been taken for at least 6 weeks at least at the minimum therapeutic dose according to British National Formulary (BNF) [26] and Maudsley prescribing guidelines [27]. Relapse whilst on an antidepressant will also count as a failed treatment trial. During the twelve weeks of active/placebo treatment patients will be requested to remain on a stable dose of the antidepressant they are taking. Participants will not be permitted to start a psychosocial intervention or psychotherapy during the study period, however those who were already engaged in these treatments at the screening stage will be permitted to continue their treatment.

Inclusion Criteria

Male and female patients aged 18 to 75 years; diagnosis of major depressive episode confirmed by SCID-5; two or more failed trials of antidepressant medication, at minimum effective doses (as per BNF and Maudsley Prescribing guidelines) for at least 6 weeks; in contact with mental health services; able to demonstrate the capacity to provide informed consent as assessed by their own clinician; able to complete the required evaluations and take oral medication; effective contraceptive precautions (either the use of barrier methods or the oral contraceptive pill) to be taken by women of child-bearing age.; a negative pregnancy test will be required in order to meet inclusion criteria.

Exclusion Criteria

Primary psychotic disorder or bipolar disorder; history of intolerance to statins or presence of any contraindication to statins; presence of any serious medical condition or neurological problem; presence of autoimmune or inflammatory disorder (e.g. systemic lupus

erythematosus, rheumatoid arthritis and inflammatory bowel disease); alcohol or drug dependence; active suicidal ideation; pregnant or breast-feeding. Patients with a serum low density lipoprotein (LDL) level of <80 mg/dL at baseline are also excluded from the study as are patients with abnormal hepatic enzymes (Aspartate transaminase (AST) and Alanine transaminase (ALT)) or abnormal Lactate Dehydrogenase (LDH) or Creatnine Phosphokinase (CPK) values at baseline. Patients already on statin treatment will also be excluded.

The criteria for leaving the study are: (1) at the participant's request; (2) at the discretion of the responsible physician or trial investigator (e.g. an adverse event, poor compliance). Poor compliance is defined as either taking <75% of study medication between assessments points at baseline, week 2, 4, 8 and 12 or missing trial medications all together for ≥ 7 days in period (3) pregnancy.

Randomisation and Allocation Concealment

Randomisation service will be provided by an independent statistical support service based in the UK. We will stratify randomization based on severity of depressive symptoms at baseline and study centre. Participants will be assigned to groups via random generation of allocation sequence, balanced across allocation group. Each participant will be assigned a unique study patient identification number once they have given informed consent and eligibility has been confirmed. The central trial pharmacist will prepare a 12-week package of treatment bearing the patient's name and ID number and send it to the site pharmacy. Thus, the site pharmacy will not know the treatment allocated to the patient. A study information leaflet will be given

to the participant, explaining that they are in a clinical trial and that in addition to TAU they are taking a placebo or simvastatin. This leaflet will also have the name of the local PI's.

Allocation will be masked from study investigators and co-investigators until participants have completed all follow-ups and the database is cleaned and locked. The trial pharmacist at the central pharmacy will keep the drug codes. There will be detailed protocols regarding maintenance of blindness for raters and governing unblinding procedures in case of emergency for pharmacists. To assess the integrity of blinding procedures, participants and independent raters will be asked to complete a conventional guess form asking whether they believe participants received simvastatin or placebo as a treatment after the final ratings have been completed.

If any participant develops side effects or suffers an emergency requiring drug unblinding, the responsible consultant and care team will be informed of the trial drug, probable side-effects and make a decision of participant continuation in the trial. Participant well being and safety will be crucial at all times and locally and internationally accepted Good Clinical Practice will be observed across the trial's governance procedures

Follow-up

Participants will continue with the treatment currently provided by their clinical teams. Simvastatin will be started at a dose of 20mg daily. Assessments will be at baseline, weeks 2, 4, 8 and 12.

The clinical team and consultant psychiatrist will continue to oversee routine care for each participant although research assistants will be contactable for the duration of the study to respond to any concerns. Demographic data will be collected by RAs at baseline and will include age, gender, marital status, socio-economic status, highest level of education, smoking status, and concomitant medication.

Participants will be asked to bring all leftover medications to each follow up visit, so that RAs can complete a pill count to monitor compliance. On each visit the research assistant will meet with patient at an agreed location to complete the follow up assessment.

Outcome measures

The primary outcome measure will be the Montgomery-Asberg Depression Rating Scale (MADRS) [28] total score at week 12. Ratings will be made on the basis of a semi-structured clinical interview at baseline and at every follow-up visit until week 12. The MADRS was chosen as the primary outcome measure as it has been demonstrated to be more precise in estimating changes in depression severity compared to other measures [29, 30]. However the HRSD remains a useful screening tool and the use of different assessment scales at screening and follow-up mitigates the risk of clinician bias whereby raters over-estimate symptom severity at screening stage to increase study recruitment. This can then mask the true treatment effect and may lead to an inflated placebo response.

Other outcomes recorded will include the 24-item Hamilton Rating Scale for Depression (HRSD-24) [25] which includes assessment of atypical depressive symptoms, the Clinical Global Impression (CGI) scale [31], an overall measure of illness severity, the 7-item

Generalized Anxiety Disorder scale (GAD-7) [32], a self-report measure of severity for generalised anxiety disorder and a modified Morisky measurement of medication adherence (MMAS-4) [33]. We will also assess changes in body mass index (BMI) from baseline to week 12. All scales have been validated for use in the Urdu language and have been used in previous studies in Pakistan [24]. Adverse effects will be monitored using a side effect assessment scale based on the product monograph of simvastatin.

Measurement of biomarkers

Participants will be asked to provide two blood samples: at baseline and week 12. We aim to analyse the relationship of simvastatin to lipid and inflammatory markers and determine if this is associated with symptom change prior to a clinical effect (if detected). The biomarkers analysed will include lipid biomarkers HDL, LDL and C-Reactive Protein (CRP), a peripheral marker of inflammation.

Inter-rater reliability

Research assistants have been trained in Structured Clinical Interview for DSM-5 (SCID) and in carrying out clinical assessments by the University of Manchester for a previous grant funded study. Inter-rater reliability will be measured using calculations of the Kappa statistic [34] assessed during investigator meeting rating precision exercises.

Statistical Analysis

Initial descriptive analysis will be conducted to study the profile of the subjects and investigate group differences at baseline on main demographics and clinical measures Frequencies and proportions will be used for categorical variables and mean and standard deviations, median and inter-quartile range, minimum and maximum for continuous variables. No statistical significance tests will be used to compare groups. Drop out, visit frequency and adherence to treatment will be summarized by group with the appropriate descriptive statistics.

The main analysis will follow the Intention-to-treat (ITT) principle in which subjects are assigned to treatment groups as randomized. Subjects that drop out of the study will be included in the analysis provided that they have a baseline score for the outcome. For the primary hypothesis, the MADRS scores at week 12 will be compared between groups using a mixed effect model. The outcome for the model will be all post-baseline MADRS scores and the fixed effects will include baseline MADRS scores, treatment group, time (post-baseline timepoints) and a treatment group by timepoint interaction. To account for the dependencies between repeated measures on the same patients, an intercept for each individual will be included as a random effect. A linear contrast will be used to test the primary hypothesis, the difference between groups in at week 12 in MADRS scores. Given the modest sample size it will likely not be possible to use as random slope for each subject, however the covariance matrix of the residuals of the model can be allowed to correlate to account for dependencies across time. Two tailed tests and an alpha level of 0.05 will be used for significance. All estimates will be presented with the 95% confidence interval. To give an estimate of effect size, standardized regression coefficients will be calculated using the overall SD of the baseline outcome scores as the metric. One advantage of this approach is it gives the magnitude and direction of effect in units of patient population of interest.

Secondary analyses will use similar mixed models to that of the primary outcome, baseline adjusted with time, treatment group and the interaction between treatment group and time as independent variables. As for the primary outcome, the main timepoint of interest is 12 week follow up and treatment effects will be determined by contrasts at the measured timepoint. To compare response and remission rates between groups logistic regression will be used in which patients will be classified as in remission or not according to standard criteria on the MADRS scale. As there are multiple measures, p values will be adjusted for multiple comparisons using a correction based on controlling the family-wise error. Outcomes at timepoints other than 12 week follow-up will be considered exploratory and the main focus will be effect sizes (standardized regression coefficients).

To compare frequency of adverse events descriptive summaries will be presented by treatment group. Differences in the rates of AEs will be assessed by generalized linear mixed model with Poission or negative binomial distribution will be used given sufficient numbers of events, where time, treatment group and their interaction are fixed effects with subjects as random effects. Alternatively, simple count models will be used at each timepoint given the limited sample size and complexity of GLMM models.

It is likely there will be some drop-out of participants from the trial. Baseline predictors of missingness can be identified using logistic regression with the outcome (missing or not). Mixed effect models fitted through maximum likelihood can use all available information in the data potentially avoiding removal of dropout subjects from the analysis. Moreover, by including baseline predictors of missingness in the statistical models, the analysis may

assume the data to be missing at random (MAR). Diagnostic analysis will be conducted through checking residuals for outliers, influential data points and normality. If outliers or influential points are found a sensitivity analysis will conducted after removing such points to see if the main findings change.

The exploratory hypothesis that baseline lipid/CRP levels will affect response to simvastatin will be tested by adding the baseline lipid/CRP to a model for the primary outcome at 12 weeks, adjusted for baseline and including a treatment group by baseline lipid/CRP. The lipid/CRP level will be considered moderators if interaction with treatment group is significant (at significance level of 0.05, using bootstrapping for inference and construction of confidence intervals). If this is the case, exploratory plots that look at the group effect at different levels of the moderators will be used to study the nature of the moderation.

In order to test the hypothesis that changes in peripheral levels of lipids/CRP will be a mediator in the path between treatment and change in MADRS scores, an initial descriptive analysis will be conducted to look at the bivariate association between treatment group and change in lipid/CRP levels, and between change in lipid/CRP (at 4 and 12 weeks) and change in MADRS scores. A mediation model will then be fitted to the data that joint models the effects of treatment on the change in lipid/CRP at 4 weeks and MADRS at 12 weeks. The mediation effect is tested by the proportion of the total effect from treatment group to change in MADRS that goes through change in lipid/CRP. This model will be fitted in Lavaan in R or Mplus 7.11[35] and the indirect effect estimated through bootstrap resampling. Given the sample size, note the effect sizes for the indirect mediation pathway would need to be relatively large [36].

Safety Monitoring

Adverse events monitoring from the time informed consent is obtained until follow-up at 12 weeks (using the side effect assessment scale). Laboratory parameters including total serum cholesterol, LDL, HDL, triglyceride, AST, ALT, LDH and CPK will be checked by the study physician before patient entry into the trial, and if there are any safety issues for which the patient should not be started on study medication, he/she will not be included to the study.

Study coordination

Local investigators will chair a weekly meeting with research assistants to help coordinate the study. The chief investigator will hold meetings with the research team every two weeks via Skype.

Data protection and confidentiality

For data security, consent forms and paper copies of assessment tools having any identifying information such as name, address or phone number of participants will all be stored in locked filing cabinets in a secure office. All computerised data will be encrypted and password protected. We will also maintain quarterly site audits of the data and security protocols across each site. These audits will be independent from the investigators and the sponsor.

Trial Steering Committee (TSC)

The responsibility of TSC will be to offer the overall supervision and monitoring of conduct of the trial. The TSC will be independent of the investigators, their employing bodies, funders and sponsors. The TSC will monitor overall trial progress, conduct and will also advise on scientific credibility. The TSC will reflect and act, as suitable, upon the recommendations of the Data Safety and Monitoring Board (DSMB) for deciding if the trial needs to be stopped on grounds of safety or efficacy.

Data Safety and Monitoring Board (DSMB)

NIH guidelines will be followed for DSMB, for monitoring and conduct of the study. The study team will be accountable to an independent DSMB, which will comprise of independent members including a chair with expertise in clinical trials, a biostatistician, one clinician with expertise in efficacy monitoring and one clinician with expertise in safety monitoring. The board will meet once a year and when necessary. The DSMB will be the only body having access to the unblinded data. The DSMB will make its recommendations to the PI and TSC.

Declaration of Helsinki

The clinical trial will be conducted according to the guidelines of the "Declaration of Helsinki" (1974) as revised in Tokyo (1975), Venice (1983), Hong Kong (1989), South Africa (1996), and Scotland (2000). The research team will comply with International Conference on Harm minimisation/Good Clinical Practice (ICHGCP) Guidelines (1996) which are in accordance of the principles of the Declaration of Helsinki.

Discussion

At least a third of patients with MDD are treatment-resistant [2] and up to a third of patients also show evidence of an activated inflammatory response [37]. There is increasing evidence linking immune-metabolic disturbances to MDD. Numerous studies have demonstrated the anti-inflammatory effects of statins in altering cytokine release and phagocytic activity [38-43]. Simvastatin has been shown to be superior to alternative statins in preventing neurodegenerative conditions, due to its permeability across the blood brain barrier and ability to prevent against cell death [44]. Moreover, treatment-resistant depression (TRD) has been shown to be a neuroprogressive condition [45] yet despite clinical trials demonstrating simvastatin's potential efficacy as an adjuvant treatment for MDD [22], we are unaware of any clinical trials in TRD.

To our knowledge, the current trial will be the first trial investigating the use of simvastatin as augmentation strategy in patients with TRD. If this study indicates that adjuvant simvastatin is efficacious in reducing depressive symptoms, it will deliver immediate clinical benefit. It is an inexpensive, off-patent drug that is readily available worldwide. It could potentially be a cost-effective treatment option in settings with diminishing resources such as low- and middle-income countries as well as other publicly funded health care services. The results of the biomarker analysis may provide insights in to the mechanism of action of simvastatin and if reductions in plasma lipids and/or inflammatory biomarkers are associated with an individual response to adjuvant simvastatin, it could inform the development of a "precision medicine" approach to the treatment of TRD. Furthermore, the results of this study will inform the current evidence base of treatment options for patients with treatment-resistant depressive symptoms, a condition that causes significant disability and functional

impairment yet for which there is a clear paucity of controlled clinical trials of novel treatments.

References:

1. World Health Organisation. Depression and Other Common Mental Disorders Global Health Estimates. 2017.
2. Rush AJ, Trivedi MH, Wisniewski SR et al. Acute and longer-term outcomes in depressed outpatients requiring one or several treatment steps: a STAR*D report. Am J Psychiatry 2006; 163:1905–17.
3. Carvalho AF, Berk M, Hyphantis TN, McIntyre RS. The integrative management of treatment-resistant depression: a comprehensive review and perspectives. Psychother Psychosom. 2014;83(2):70-88. doi: 10.1159/000357500. Epub 2014 Jan 22.
4. Rosenblat JD, McIntyre RS, Alves GS, Fountoulakis KN, Carvalho AF. Beyond Monoamines-Novel Targets for Treatment-Resistant Depression: A Comprehensive Review. Curr Neuropharmacol. 2015;13(5):636-55.
5. Ludka FK, Constantino LC2, Kuminek G, Binder LB, Zomkowski AD, Cunha MP et al. Atorvastatin evokes a serotonergic system-dependent antidepressant-like effect in mice. Pharmacol Biochem Behav. 2014 Jul;122:253-60. doi: 10.1016/j.pbb.2014.04.005. Epub 2014 Apr 21.
6. Köhler O, Gasse C, Petersen L, Ingstrup KG, Nierenberg AA, Mors O et al. The Effect of Concomitant Treatment With SSRIs and Statins: A Population-Based Study. Am J Psychiatry. 2016 Aug 1;173(8):807-15. doi: 10.1176/appi.ajp.2016.15040463. Epub 2016 May 3.

7. Parsaik AK, Singh B, Murad MH, Singh K, Mascarenhas SS, Williams MD et al. Statins use and risk of depression: a systematic review and meta-analysis. J Affect Disord. 2014 May;160:62-7. doi: 10.1016/j.jad.2013.11.026. Epub 2013 Dec 17.
8. Salagre E, Fernandes BS, Dodd S, Brownstein DJ, Berk, M. Statins for the treatment of depression: A metaanalysis of randomized, double-blind, placebo-controlled trials. Journal of Affective Disorders, 2016;200: 235-42.
9. Köhler-Forsberg O, Gasse C, Berk M, Østergaard SD. Do Statins Have Antidepressant Effects? CNS Drugs. 2017 May;31(5):335-343. doi: 10.1007/s40263-017-0422-3.
10. McFarland AJ, Anoopkumar-Dukie S, Arora DS, Grant GD, McDermott CM, Perkins AV et al. Molecular mechanisms underlying the effects of statins in the central nervous system. Int J Mol Sci. 2014 Nov 10;15(11):20607-37. doi: 10.3390/ijms151120607.
11. Herbet M, Izdebska M, Piątkowska-Chmiel I, Poleszak E, Jagiełło-Wójtowicz E. Estimation of oxidative stress parameters in rats after simultaneous administration of rosuvastatin with antidepressants. Pharmacol Rep. 2016 Feb;68(1):172-6. doi: 10.1016/j.pharep.2015.08.004. Epub 2015 Aug 20.
12. Moylan S, Berk M, Dean OM, Samuni Y, Williams LJ, O'Neil A et al. Oxidative & nitrosative stress in depression: why so much stress? Neurosci Biobehav Rev. 2014 Sep;45:46-62. doi: 10.1016/j.neubiorev.2014.05.007. Epub 2014 May 21.
13. Köhler CA, Freitas TH, Maes M, de Andrade NQ, Liu CS, Fernandes BS et al. Peripheral cytokine and chemokine alterations in depression: a meta-analysis of 82 studies. Acta Psychiatr Scand. 2017 May;135(5):373-387. doi: 10.1111/acps.12698. Epub 2017 Jan 25.

14. Köhler CA, Freitas TH, Stubbs B, Maes M, Solmi M, Veronese N et al. Peripheral Alterations in Cytokine and Chemokine Levels After Antidepressant Drug Treatment for Major Depressive Disorder: Systematic Review and Meta-Analysis. Mol Neurobiol. 2018 May;55(5):4195-4206. doi: 10.1007/s12035-017-0632-1. Epub 2017 Jun 13.

15. Parekh A, Smeeth D, Milner Y, Thuret S. The Role of Lipid Biomarkers in Major Depression. Healthcare. 2017; 5: 5.

16. Lamers F, Milaneschi Y, de Jonge P, Giltay EJ, Penninx BWJH. Metabolic and inflammatory markers: associations with individual depressive symptoms. Psychol Med. 2018 May;48(7):1102-1110. doi: 10.1017/S0033291717002483. Epub 2017 Sep 11.

17. Stewart JW, McGrath PJ, Fava M, Wisniewski SR, Zisook S, Cook I et al. Do atypical features affect outcome in depressed outpatients treated with citalopram? The International Journal of Neuropsychopharmacology. 2010;13:15–30.

18. Johnson-Anuna LN, Eckert GP, Keller JH, Igbavboa U, Franke C, Fechner T et al. Chronic administration of statins alters multiple gene expression patterns in mouse cerebral cortex. J. Pharmacol. Exp. Ther. 2005;312:786–793.

19. Thelen KM, Rentsch KM, Gutteck U, Heverin M, Olin M, Andersson U et al. Brain cholesterol synthesis in mice is affected by high dose of simvastatin but not of pravastatin. J Pharmacol Exp Ther. 2006;316:1146–1152.

20. Ma, K., Zhang H., Baloch Z. Pathogenetic and Therapeutic Applications of Tumor Necrosis Factor-α (TNF-α) in Major Depressive Disorder: A Systematic Review. Int. J. Mol. Sci. 2016; 17: 733–753.

21. Liu Y, Ho RC, Mak A. Interleukin (IL)-6, tumour necrosis factor alpha (TNF-α) and soluble interleukin-2 receptors (sIL-2R) are elevated in patients with major depressive disorder: a meta-analysis and metaregression. J. Affect. Disord. 2012; 139: 230–239.
22. Gougol A, Zareh-Mohammadi N, Raheb S, Farokhnia M, Salimi S, Iranpour N et al. Simvastatin as an adjuvant therapy to fluoxetine in patients with moderate to severe major depression: a double-blind placebo-controlled trial. J.Psychopharmacol. 2015;29: 575–581.
23. First MB, Williams JBW, Karg RS, Spitzer RL: Structured Clinical Interview for DSM-5—Research Version (SCID-5 for DSM-5, Research Version; SCID-5-RV). Arlington, VA, American Psychiatric Association, 2015
24. Husain MI, Chaudhry IB, Husain N, Khoso AB, Rahman RR, Hamirani MM et al. Minocycline as an adjunct for treatment-resistant depressive symptoms: A pilot randomised placebo-controlled trial. J Psychopharmacol. 2017 Sep;31(9):1166-1175. doi: 10.1177/0269881117724352. Epub 2017 Aug 31.
25. Hamilton M. Rating depressive patients. J Clin Psychiatry. 1980 Dec;41(12 Pt 2):21-4.
26. Joint Formulary Committee. British National Formulary. London: BMJ Group and Pharmaceutical Press; 2017.
27. Taylor D, Barnes TRE, Young AH. The Maudsley Prescribing Guidelines, Thirteenth Edition. London: CRC Press; 2018.
28. Montgomery SA, Asberg A. A new depression scale designed to be sensitive to change.The British Journal of Psychiatry. 1979; 134: 382-389.
29. Carmody T, Rush AJ, Bernstein I, et al. The Montgomery Äsberg and the Hamilton Ratings of Depression: A Comparison of Measures. Eur Neuropsychopharmacol. 2006 Dec; 16(8): 601–611.

30. Carmody TJ, Rush AJ, Bernstein I, Warden D, Brannan S, Burnham D et al. The Montgomery Asberg and the Hamilton ratings of depression: a comparison of measures. Eur Neuropsychopharmacol. 2006 Dec;16(8):601-11. Epub 2006 Jun 12.
31. Busner J, Targum SD. The Clinical Global Impressions Scale. Psychiatry (Edgmont) 2007, Jul; 4(7): 28–37.
32. Spitzer RL, Kroenke K, Williams JB, et al; A brief measure for assessing generalized anxiety disorder: the GAD-7. Arch Intern Med. 2006 May 22; 166(10):1092-7.
33. Morisky DE, Green LW, Levine DM. Concurrent and predictive validity of a self-reported measure of medication adherence and long-term predictive validity of blood pressure control. Med Care. 1986; 24:67–74.
34. Fleiss JL. Measuring nominal scale agreement among many raters. Psychological Bulletin, 1971; 76(5):378–382.
35. Muthén, L.K. and Muthén, B.O. (1998-2012). Mplus User's Guide. Seventh Edition. Los Angeles, CA: Muthén & Muthén
36. Fritz, M. S., & MacKinnon, D. P. (2007). Required Sample Size to Detect the Mediated Effect. *Psychological Science*, *18*(3), 233–239. https://doi.org/10.1111/j.1467-9280.2007.01882.x
37. Wium-Andersen MK, Orsted DD, Nielsen SF et al. Elevated C-reactive protein levels, psychological distress, and depression in 73, 131 individuals. JAMA Psychiatry 2013; 70: 176-184.
38. Benati D, Ferro M, Savino MT, Ulivieri C, Schiavo E, Nuccitelli A, Pasini FL, Baldari CT. Opposite effects of simvastatin on the bactericidal and inflammatory response of macrophages to opsonized S. aureus. J Leukoc Biol. 2009, 87: 433-442.

39. Loike JD. Statin Inhibition of Fc Receptor-Mediated Phagocytosis by Macrophages Is Modulated by Cell Activation and Cholesterol. Arterioscler Thromb Vasc Biol. 2004, 24: 2051-2056.

40. Muniz-Junqueira MI, Karnib SR, de Paula-Coelho VN, Junqueira LF. Effects of pravastatin on the in vitro phagocytic function and hydrogen peroxide production by monocytes of healthy individuals. Int Immunopharmacol. 2006, 6: 53-60.

41. Salman H, Bergman M, Djaldetti M, Bessler H. Hydrophobic but not hydrophilic statins enhance phagocytosis and decrease apoptosis of human peripheral blood cells in vitro. Biomed Pharmacother. 2008, 62: 41-45.

42. Tanaka N, Abe-Dohmae S, Iwamoto N, Fitzgerald ML, Yokoyama S. HMG-CoA reductase inhibitors enhance phagocytosis by upregulating ATP-binding cassette transporter A7. Atherosclerosis. 2011, 217: 407-414.

43. Djaldetti M, Salman H, Bergman M, Bessler H. Effect of pravastatin, simvastatin and atorvastatin on the phagocytic activity of mouse peritoneal macrophages. Exp Mol Pathol. 2006, 80: 160-164.

44. Sierra S, Ramos MC, Molina P, Esteo C, Vázquez JA, Burgos JS. Statins as neuroprotectants: a comparative in vitro study of lipophilicity, blood-brain-barrier penetration, lowering of brain cholesterol, and decrease of neuron cell death. J. Alzheimers Dis., 23 (2011), pp. 307–318

45. Setiawan E, Attwells S, Wilson AA, Mizrahi R, Rusjan PM, Miler L et al. Association of translocator protein total distribution volume with duration of untreated major depressive disorder: a cross-sectional study. Lancet Psychiatry. 2018 Apr;5(4):339-347. doi: 10.1016/S2215-0366(18)30048-8. Epub 2018 Feb 26.

Figure 1: Flowchart of randomised controlled trial

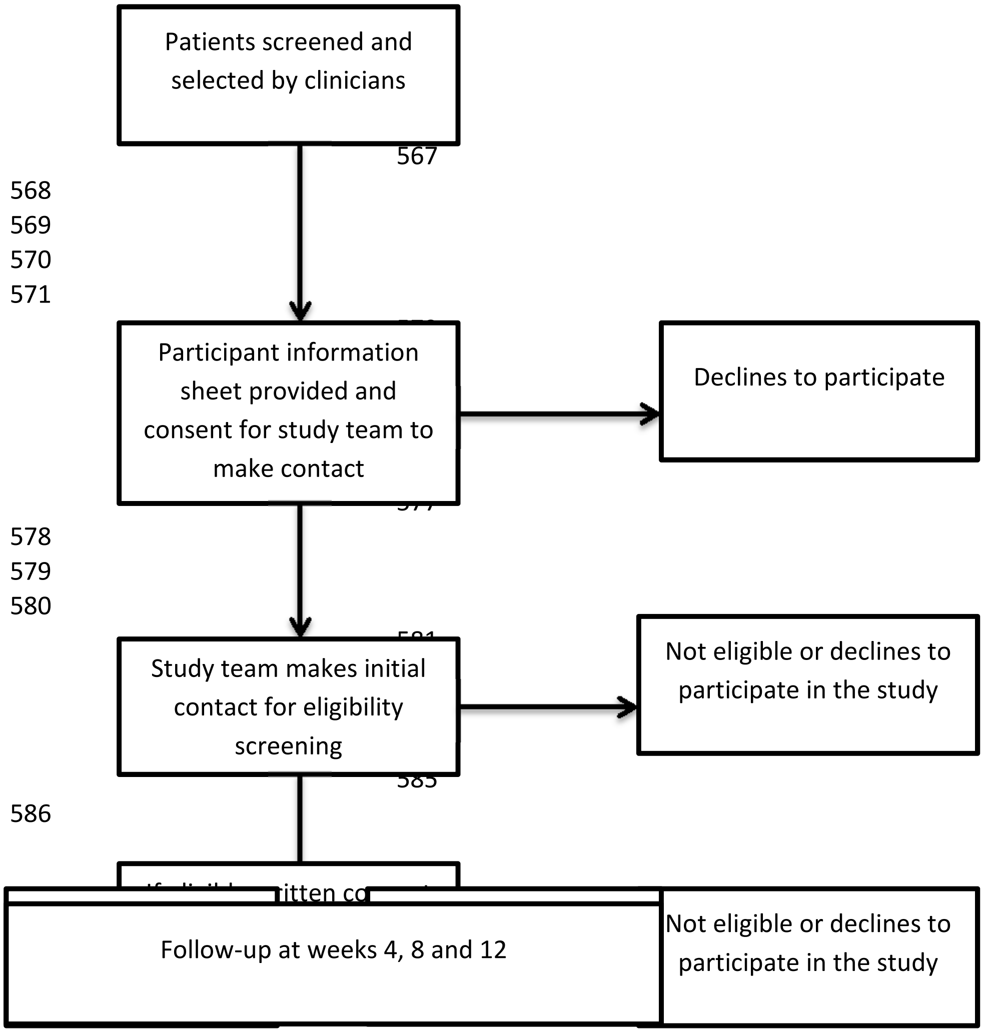

Table 1: Assessment Schedule

Assessment	Baseline	Week 2	Week 4	Week 8	Week 12	Ad hoc
Medical Confirmation of Eligibility, SCID-5	✓					
Randomisation	✓					
MADRS	✓	✓	✓	✓	✓	
HRSD-24	✓	✓	✓	✓	✓	
CGI	✓	✓	✓	✓	✓	
GAD-7	✓	✓	✓	✓	✓	
MMAS-4	✓	✓	✓	✓	✓	
Simvastatin dispensed	✓	✓	✓	✓	✓	
TAU/Concomitant medication	✓	✓	✓	✓	✓	
Adverse Effects Scale		✓	✓	✓	✓	✓
Basic clinical & psychiatric information	✓					
BMI	✓	✓	✓	✓	✓	
Biomarkers (CRP, lipids)	✓		✓		✓	

Telephone contact						✓
