# Supplementary material for: Effect of Adjunctive Simvastatin on Depressive Symptoms Among Adults With Treatment-Resistant Depression: A Randomized Clinical Trial
Source: JAMA Netw Open. 2023 Feb 20;6(2):e230147. doi: 10.1001/jamanetworkopen.2023.0147 (PMC9941891; doi:10.1001/jamanetworkopen.2023.0147)
Supplement: Supplement 2. — eTable 1. Mean Group Difference Between Slopes for Baseline MADRS Scores per Visit eTable 2. Mean Group Difference Between Slopes for Baseline BMI Scores per Visit eTable 3. Mean Group Difference Between Slopes for Baseline CRP Scores per Visit eTable 4. Mean Group Difference Between Slopes for Baseline HDL Scores per Visit eTable 5. Mean Group Difference Between Slopes for Baseline LDL Scores per Visit eTable 6. Estimated Means and 95% CI for Mediation Model With CRP (at 12 Weeks) as Mediator eTable 7. Estimated Means and 95% CI for Mediation Model With log(HDL) (at 12 Weeks) as Mediator eTable 8. Estimated Means and 95% CI for Mediation Model With log(LDL) (at 12 Weeks) as Mediator eFigure 1. Estimated Mean Slopes and 95% CI for Baseline (Log) LDL vs Outcome by Group and Study Visit eFigure 2. Frequency of Adverse Effects Across Treatment Groups eFigure 3. Clinical Global Impression Outcomes Across Treatment Groups [file jamanetwopen-e230147-s002.pdf]

Supplementary Online Content

Husain MI, Chaudhry IB, Khoso AB, et al. Effect of adjunctive simvastatin on depressive symptoms among adults with treatment-resistant depression: a randomized clinical trial. *JAMA Netw Open*. 2023;6(2):e230147.
doi:10.1001/jamanetworkopen.2023.0147

eTable 1. Mean Group Difference Between Slopes for Baseline MADRS Scores per Visit
eTable 2. Mean Group Difference Between Slopes for Baseline BMI Scores per Visit
eTable 3. Mean Group Difference Between Slopes for Baseline CRP Scores per Visit
eTable 4. Mean Group Difference Between Slopes for Baseline HDL Scores per Visit
eTable 5. Mean Group Difference Between Slopes for Baseline LDL Scores per Visit
eTable 6. Estimated Means and 95% CI for Mediation Model With CRP (at 12 Weeks) as Mediator
eTable 7. Estimated Means and 95% CI for Mediation Model With log(HDL) (at 12 Weeks) as Mediator
eTable 8. Estimated Means and 95% CI for Mediation Model With log(LDL) (at 12 Weeks) as Mediator
eFigure 1. Estimated Mean Slopes and 95% CI for Baseline (Log) LDL vs Outcome by Group and Study Visit
eFigure 2. Frequency of Adverse Effects Across Treatment Groups
eFigure 3. Clinical Global Impression Outcomes Across Treatment Groups

This supplementary material has been provided by the authors to give readers additional information about their work.

© 2023 Husain MI et al. *JAMA Network Open.*

eTable 1. Mean Group Difference Between Slopes for Baseline MADRS Scores per Visit

**Baseline Severity: Simvastatin - Placebo**		
**Visit**	**Difference in slopes (95% CI)**	**p**
Week 2	0.11 (-0.31, 0.53)	0.602
Week 4	-0.18 (-0.61, 0.25)	0.408
Week 8	0.05 (-0.37, 0.48)	0.810
Week 12	-0.3 (-0.73, 0.14)	0.177

eTable 2. Mean Group Difference Between Slopes for Baseline BMI Scores per Visit

**Baseline BMI: Simvastatin - Placebo**		
**Visit**	**Difference in slopes (95% CI)**	**p**
Week 2	-0.15 (-0.76, 0.47)	0.639
Week 4	0.42 (-0.2, 1.04)	0.187
Week 8	-0.18 (-0.8, 0.44)	0.575
Week 12	-0.12 (-0.75, 0.51)	0.707

eTable 3. Mean Group Difference Between Slopes for Baseline CRP Scores per Visit

**Baseline CRP: Simvastatin - Placebo**		
**Visit**	**Difference in slopes (95% CI)**	**p**
Week 2	0.12 (-2.94, 3.18)	0.938
Week 4	-2.16 (-5.22, 0.91)	0.167
Week 8	-0.96 (-4.02, 2.11)	0.539
Week 12	-2.74 (-5.81, 0.32)	0.079

eTable 4. Mean Group Difference Between Slopes for Baseline HDL Scores per Visit

**Baseline HDL: Simvastatin - Placebo**		
**Visit**	**Difference in slopes (95% CI)**	**p**
Week 2	1.72 (-1.53, 4.96)	0.299
Week 4	0.86 (-2.4, 4.12)	0.604
Week 8	0.39 (-2.86, 3.65)	0.812
Week 12	1.54 (-1.72, 4.8)	0.353

eTable 5. Mean Group Difference Between Slopes for Baseline LDL Scores per Visit

**Baseline LDL: Simvastatin - Placebo**		
**Visit**	**Difference in slopes (95% CI)**	**p**
2	3.34 (0.35, 6.32)	0.028
4	2.89 (-0.12, 5.91)	0.060
8	2.35 (-0.65, 5.35)	0.125
12	4.58 (1.58, 7.58)	0.003

eTable 6. Estimated Means and 95% CI for Mediation Model With CRP (at 12 Weeks) as Mediator

Mediation: CRP			
**Path**	**Estimate (95% CI)**	**SES (95% CI)**	**p**
Indirect Path: (a x b)	0.163 (-0.932, 1.03)	0.009 (-0.04, 0.058)	0.734
Direct Path: (c)	-1.377 (-4.412, 1.542)	-0.073 (-0.221, 0.075)	0.350
Total Effect: (axb) + c	-1.214 (-4.536, 1.582)	-0.065 (-0.219, 0.09)	0.428
a Path	-0.026 (-0.163, 0.136)	-0.013 (-0.087, 0.061)	0.729
b Path	-6.237 (-10.209, -0.566)	-0.657 (-1.12, -0.195)	0.018

© 2023 Husain MI et al. *JAMA Network Open.*

eTable 7. Estimated Means and 95% CI for Mediation Model With log(HDL) (at 12 Weeks) as Mediator

Mediation: HDL			
**Path**	**Estimate (95% CI)**	**SES (95% CI)**	**p**
Indirect Path: (a x b)	-0.089 (-0.635, 0.508)	-0.005 (-0.022, 0.013)	0.731
Direct Path: (c)	-1.082 (-4.407, 1.889)	-0.058 (-0.221, 0.106)	0.501
Total Effect: (axb) + c	-1.172 (-4.489, 1.986)	-0.062 (-0.228, 0.103)	0.467
a Path	0.032 (-0.044, 0.111)	0.068 (-0.097, 0.233)	0.419
b Path	-2.818 (-11.928, 7.437)	-0.07 (-0.311, 0.172)	0.576

eTable 8. Estimated Means and 95% CI for Mediation Model With log(LDL) (at 12 Weeks) as Mediator

Mediation: LDL			
**Path**	**Estimate (95% CI)**	**SES (95% CI)**	**p**
Indirect Path: (a x b)	0.063 (-0.236, 0.715)	0.003 (-0.015, 0.022)	0.785
Direct Path: (c)	-1.42 (-4.653, 1.522)	-0.076 (-0.239, 0.088)	0.375
Total Effect: (axb) + c	-1.357 (-4.533, 1.465)	-0.072 (-0.234, 0.09)	0.389
a Path	-0.021 (-0.126, 0.062)	-0.035 (-0.192, 0.122)	0.664
b Path	-2.955 (-8.752, 3.997)	-0.096 (-0.302, 0.11)	0.363

eFigure 1. Estimated Mean Slopes and 95% CI for Baseline (Log) LDL vs Outcome by Group and Study Visit

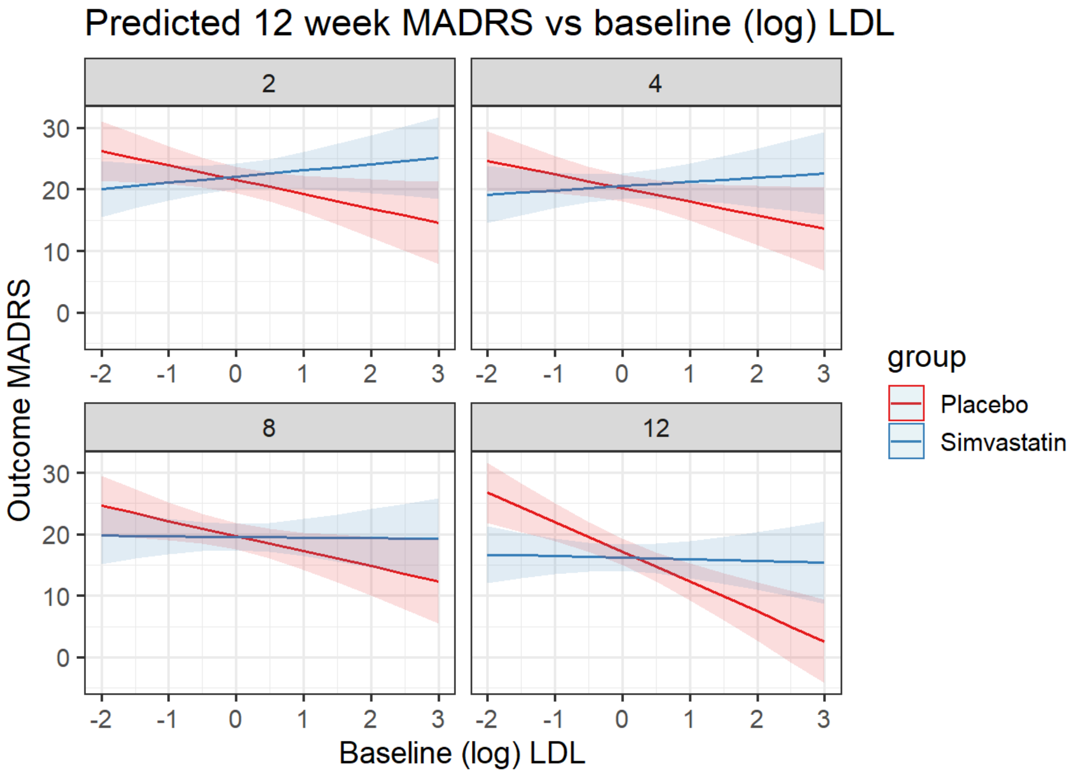

© 2023 Husain MI et al. *JAMA Network Open.*

eFigure 2. Frequency of Adverse Effects Across Treatment Groups

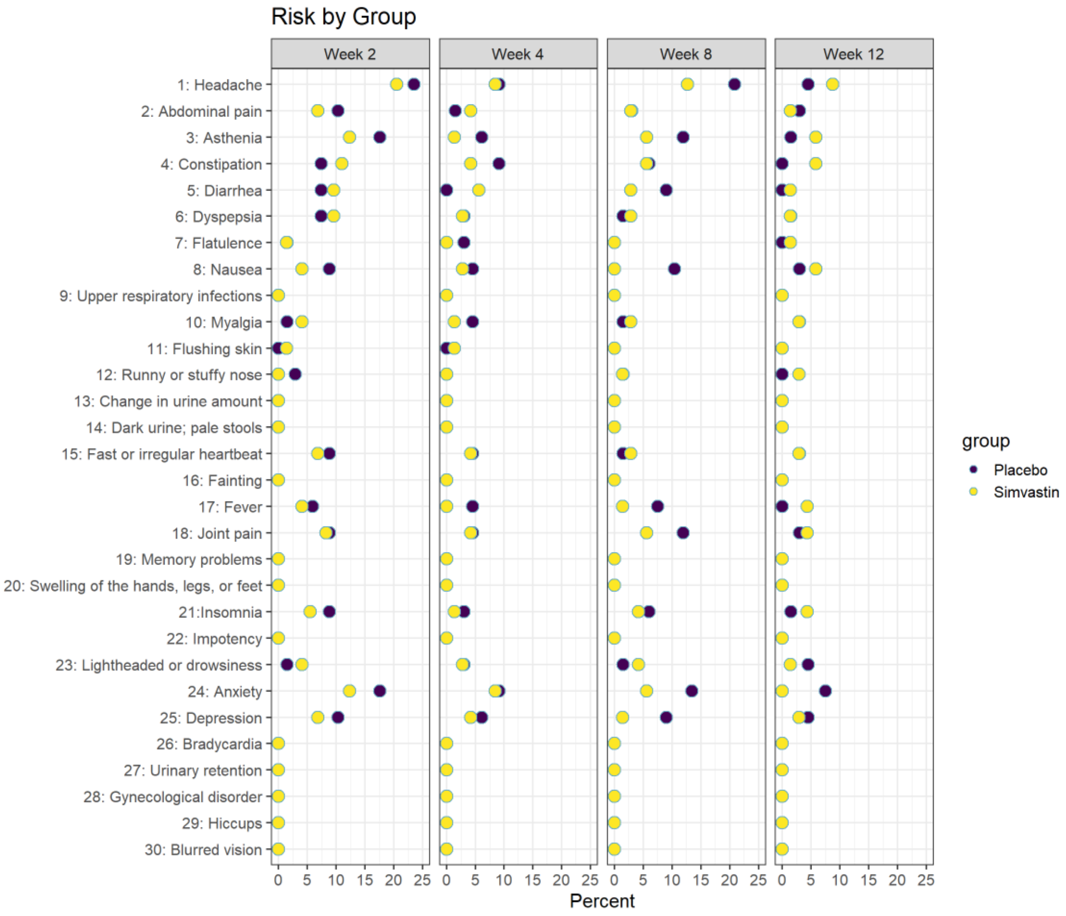

eFigure 3. Clinical Global Impression Outcomes Across Treatment Groups

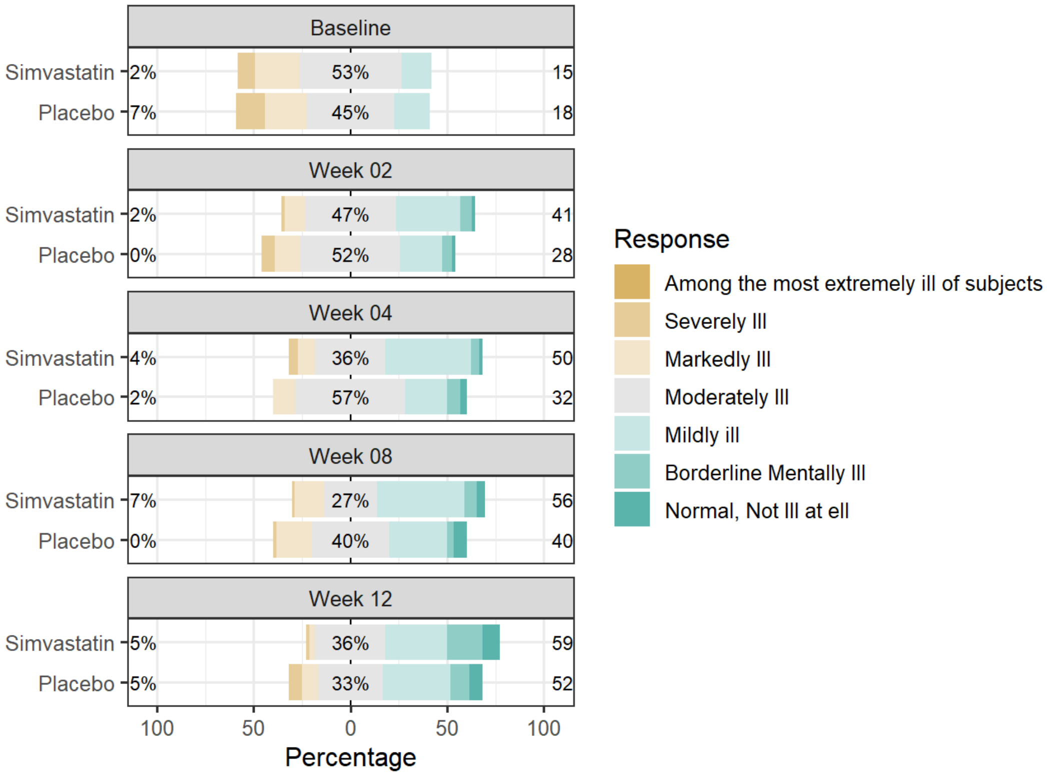

© 2023 Husain MI et al. *JAMA Network Open.*
