# Supplementary material for: Effect of Adjunctive Simvastatin on Depressive Symptoms Among Adults With Treatment-Resistant Depression: A Randomized Clinical Trial
Source: JAMA Netw Open. 2023 Feb 20;6(2):e230147. doi: 10.1001/jamanetworkopen.2023.0147 (PMC9941891; doi:10.1001/jamanetworkopen.2023.0147)
Supplement: Supplement 3. — Data Sharing Statement [file jamanetwopen-e230147-s003.pdf]

Data Sharing Statement

Husain. Effect of Adjunctive Simvastatin on Depressive Symptoms Among Adults With Treatment-Resistant Depression. *JAMA Netw Open*. Published February 20, 2023. doi:10.1001/jamanetworkopen.2023.0147

Data
Data available: Yes
Data types: Deidentified participant data
How to access data: ishrat.husain@camh.ca
When available: With publication

Supporting Documents
Document types: None

Additional Information
Who can access the data: Researchers whose proposed use of the data has been approved.
Types of analyses: For a specified purpose.
Mechanisms of data availability: After approval of a proposal.
